# Oral findings and comprehensive dental management of Moebius syndrome: a systematic review

**DOI:** 10.1186/s12903-024-03968-6

**Published:** 2024-02-06

**Authors:** Mario Alberto Alarcón-Sánchez, Selenne Romero-Servin, Lazar Yessayan, Seyed Ali Mosaddad, Artak Heboyan

**Affiliations:** 1 Biomedical Science, Faculty of Chemical-Biological Science, Autonomous University of Guerrero, Chilpancingo 39090, Guerrero Mexico; 2 Resident Student, Oral and Maxillofacial Pathology, National School of Higher Studies, Leon Unit of the National Autonomous University of Mexico, Leon 37684, Guanajuato Mexico; 3 Department of Therapeutic Stomatology, Faculty of Stomatology, Yerevan State Medical University after Mkhitar Heratsi, Yerevan, Armenia; 4 Student Research Committee, School of Dentistry, Shiraz University of Medical Sciences, Qasr-e-Dasht Street, Shiraz, Iran; 5 Department of Prosthodontics, Faculty of Stomatology, Yerevan State Medical University after Mkhitar Heratsi, Str. Koryun 2, Yerevan, 0025 Armenia; 6 Department of Research Analytics, Saveetha Dental College and Hospitals, Saveetha Institute of Medical and Technical Sciences, Saveetha University, Chennai, India

**Keywords:** Moebius syndrome, Oral and extraoral manifestation, Dental management, Oral complications

## Abstract

**Background:**

Moebius syndrome (MS) is a rare, non-progressive, neuromuscular, congenic disease involving the oral maxillofacial region. The present study aimed to describe the oral and extraoral findings in MS patients and their comprehensive dental management.

**Methods:**

A digital search was carried out in PubMed/MEDLINE, Scopus, Web of Science, and Google Scholar, restricted to articles in English from Jan 01, 2000, to Apr 02, 2023, following PRISMA guidelines. The methodological quality of the studies was evaluated following the JBI guidelines. Qualitative analysis was carried out on the overall result, extraoral and intraoral manifestations, considering dental management as appropriate.

**Results:**

Twenty-three studies were included, and a total of 124 cases of patients with MS were analyzed. The 82% of patients with MS were younger than 15 years of age. The most frequent extraoral manifestations were blinking and visual problems (78,22%), malformations of the upper and lower limbs (58,22%), bilateral facial paralysis (12,90%), lack of facial expression (12.09%), and unilateral facial paralysis (6,45%). On the other hand, the most frequent oral manifestations were tongue deformities (78,22%), micrognathia (37,90%), labial incompetence (36,29%), cleft palate (22,87%), gothic palate (16,12%), microstomia (15,32%), anterior open bite (15,32%), dental caries (8,87%), and periodontal disease (8,06%). The majority of MS patients were treated by pediatric dentistry (60,86%), using a surgical approach (56,52%), and orthodontic and orthopedic maxillary (43,47%) followed by restorative (39,13%), and periodontal treatments (21,73%).

**Conclusions:**

This systematic review demonstrates that patients with MS present a wide variety of oral and extraoral manifestations, for which dental treatments are planned and tailored to each patient in accordance with oral manifestations. These treatments encompass problem resolution and oral health maintenance, incorporating recent techniques in managing and treating patients with MS.

## Background

Moebius syndrome (MS) is a rare, non-progressive, neuromuscular condition present at birth [1]. This syndrome is characterized by a paralysis of the abducens (VI) and facial (VII) cranial nerves, with frequent asymmetric presentations. It may be unilateral or bilateral, partial or total, symmetrical or asymmetrical. It may also present with additional manifestations arising from disrupting other cranial nerves, such as craniofacial and orolingual malformations [2]. This rare neurological disorder is estimated to occur in 1/100,000 live births and has no gender predilection [3]. In 1888, the German ophthalmologist Paul Julius Möbius studied and classified these patients for the first time, giving rise to the name [1, 3]. The etiopathogenesis of MS is still unclear; however, two main theories have been proposed. The first theory is the ischemic type, which is due to an interruption in the vascular supply of the brainstem resulting in ischemia of the nuclei of the VII cranial nerve due to a genetic, ambient, or mechanical cause. The second theory also involves a defect in embryological development (in the rhombomere segments and nuclei of the affected nerves); however, both suggest that teratogenicity is an important etiologic factor [4]. Mutations in the MBS1, MBS2, and MBS3 gene loci have also contributed to its development through several pathways. Genes of the HOX family have also been implicated [5].

The clinical presentation of MS depends on the extent of the paralysis and the anatomical structures involved [6]. Classic MS is characterized by bilateral or unilateral paralysis of cranial nerves VI and VII; however, it can also be associated with other anomalies or syndromes, such as Kallman, Hanhart, or Poland syndrome. In some cases, patients may have a more extensive involvement affection the cranial nerves: Oculomotor (III), pathetic (IV), trigeminal (V), glossopharyngeal (IX), vagus (X), and hypoglossal (XII) [7]. The most frequent clinical manifestations of the oral and maxillofacial region included inexpressive facies (lack of smile and facial expression), low implantation of the pinna, and deformity of the ears with hearing loss, micrognathia, microstomia, cleft palate, bifid uvula, occlusal problems [8]. Dysfunctions in the temporomandibular joint have also been described, such as reduced lateralization, protrusion, and maximum opening movements [9]. The treatment of these patients is multidisciplinary and requires several specialists in the health area, such as neurologists, pediatricians, ophthalmologists, psychiatrists, and geneticists, as well as general dentists, maxillofacial surgeons, orthodontists, periodontists, among others [10]. MS patients face numerous daily challenges, including maintaining good oral health. Lack of adequate dental care can have a negative impact on these patients’ overall health. Therefore, this review focuses on the integral dental management of patients with MS, emphasizing the treatment of oral manifestations and associated extraoral complications to improve patient’s quality of life [11, 12].

In conclusion, this research aims to describe the oral and extraoral findings of patients with MS. The present study aimed to describe the oral and extraoral findings of MS patients and their comprehensive dental management. Furthermore, the study seeks to raise awareness regarding the significance of dental care for this disability.

## Methods

### Protocol development

For the literature search and selection of studies, the present work was constructed following the Preferred Reporting Items for Systematic Review and Meta-Analysis (PRISMA) guidelines [13]. The protocol was registered at the Open Science Framework (Registration DOI: 10.17605/OSF.IO/HRJSV).

### Review question

The electronic databases PubMed/MEDLINE, Scopus, Web of Science, and Google Scholar were consulted to investigate all available evidence on studies describing the comprehensive dental management of patients with Moebius Syndrome. For this purpose, the Boolean terms “OR” and “AND” were used together with search header terms (MeSH). We organized the search and selection of studies following the SPIDER question format adopted from the PICO tool. Sample (S); Moebius syndrome patients; Phenomena of interest (PI); Oral and extraoral manifestations, Design (D); clinical case reports, Evaluation (E); dental management and Research type (R); qualitative. Thus, the following research question was formulated: What are Moebius syndrome’s most frequent oral and extraoral findings, with the sub-question: What is the comprehensive dental management for these conditions?

### Eligibility criteria

Before the screening phase, the following characteristics were considered to select the best articles related to this research topic. Articles only in English language, articles published after Jan 01, 2000, in peer-reviewed journals, clinical studies, case series, and case reports, confirmed MS cases with sufficient clinical information for definitive diagnosis, and research focused on the dental management of the patient with MS, including treatment of the clinical oral manifestations. Book chapters, editorials, and short communications were excluded.

### Search strategy and study selection

The search was limited to case reports and case series only. A combination of keywords was used, including “Moebius syndrome,” “Moebius syndrome and Oral Health,” “Dental Treatment of Moebius Syndrome,” and “Moebius syndrome and Dentistry”. Searches in different databases were conducted from Jan 01, 2000, to Apr 02, 2023. The electronic search was enriched by an iterative hand search in journals related to oral pathology and medicine, maxillofacial surgery, and oral prosthetics and implantology. The journals were as follows: *“Journal of Oral Pathology & Medicine,” “Oral Surgery Oral Medicine Oral Pathology Oral Radiology,” Medicina Oral Patología Oral y Cirugía Bucal,” “Journal of Stomatology Oral and Maxillofacial Surgery,” International Journal of Oral & Maxillofacial Implants,” Journal of Oral and Maxillofacial Surgery,” British Journal of Oral & Maxillofacial Surgery,” “Oral and Maxillofacial Surgery Clinics of North America,” Journal of Cranio-Maxillofacial Surgery” and “Dentomaxillofacial Radiology.”* Table 1 shows the search strategy employed.

**Table 1:** The full search strategy in PubMed, Scopus, and Web of Science

**PubMed**	(“Mobius Syndrome/classification“[Mesh] OR “Mobius Syndrome/complications“[Mesh] OR “Mobius Syndrome/diagnosis“[Mesh] OR “Mobius Syndrome/embryology“[Mesh] OR “Mobius Syndrome/epidemiology“[Mesh] OR “Mobius Syndrome/etiology“[Mesh] OR “Mobius Syndrome/genetics“[Mesh] OR “Mobius Syndrome/history“[Mesh] OR “Mobius Syndrome/immunology“[Mesh] OR “Mobius Syndrome/microbiology“[Mesh] OR “Mobius Syndrome/pathology“[Mesh] OR “Mobius Syndrome/physiopathology“[Mesh] OR “Mobius Syndrome/prevention and control“[Mesh] OR “Mobius Syndrome/rehabilitation“[Mesh] OR “Mobius Syndrome/surgery“[Mesh] OR “Mobius Syndrome/therapy“[Mesh] )
**Scopus and Web of Science**	TITLE-ABS-KEY (Moebius syndrome AND Oral manifestations AND Dental treatment OR Oral Health)

Initially, the selection of studies was made considering the title and abstract of the articles; any ambiguity in these sections was resolved by resorting to full-text articles. The articles found in the databases were subjected to a second review according to the eligibility criteria. If any conflict arose between the principal investigators (M.A.A.S and S.R.S), a third investigator (A.H) was consulted to resolve the debate.

### Quality assessment, data extraction, and statistical analysis

The quality of the studies was assessed following the guidelines (http://jbi.global/critical-appraisal-tools) in the individual sections of case reports and case series [14]. All included articles underwent independent quality assessment by two investigators (M.A.A.S and S.R.S). The tool is based on a series of questions grouped according to the type of studies included in the systematic review that can be rated as “Yes,” “No,” “Unclear,” or “Not applicable.” According to the assessment instrument, the risk of bias was classified as high when the study reached up to 49% of the “Yes” scores, moderate from 50 to 69%, and low when it reached above 70%.

One reviewer (A.H.) performed data extraction from the previously selected articles. All relevant information such as Year of publication, first author, country, number of cases, study design, age, gender, oral and extraoral manifestation, dental treatment, dental discipline, and follow-up period were extracted and recorded first in a standardized Excel datasheet, and then in a database in the statistical program STATA V15 (Stata Corp, College Station, TX, EE.UU.). Finally, the selected articles were analyzed by descriptive statistics representing the data with mean ± standard deviation (DE), range (minimum-maximum), absolute and relative frequency. All the data were taken together to construct the systematic review.

## Results

### Study selection

**Fig. 1:**
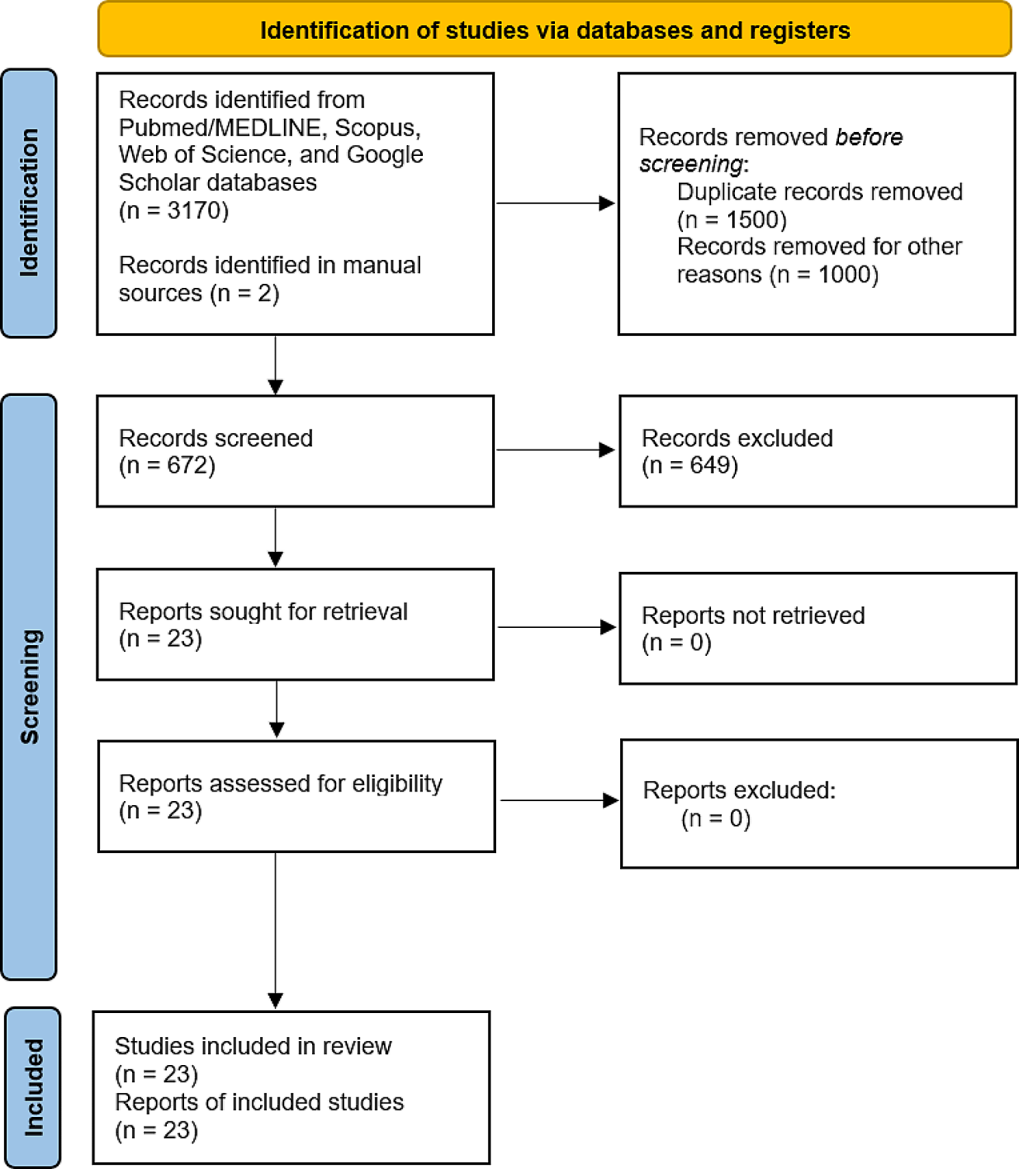
PRISMA flow diagram. PRISMA: Preferred Reporting Items for Systematic and Meta-Analyses

Initially, 3,170 articles were found in four databases, including PubMed (from which 320 articles were found), Scopus (from which 100 articles were found), Web of Science (from which another 100 articles were found), Google Scholar (from which 2,650 articles were found), and two other articles in manual sources. Three thousand one hundred seventy-two articles were obtained, reduced to 672 after eliminating duplicates and for other reasons. In the next phase, by reading titles and abstracts, the two reviewers (M.A.A.S and A.H) could exclude 649 more articles that did not agree with the research criteria and were not open access. Thus, 23 articles were considered eligible at the eligibility stage. The full texts were then read and analyzed. No articles were excluded. Therefore, 23 articles were included in this systematic review (Fig. 1).

## Study characteristics

In this study, 23 investigations were reviewed, of which 18 (78%) were case reports and 5 (22%) were case series. The total number of patients studied in the included investigations was 124, all with MS, a description of its oral and extraoral manifestations, and comprehensive dental management. Most of the articles were published after 2012 (15:65,2%). The clinical studies were published in 12 different countries. Five (21,7%) were conducted in Brazil [15–19], three (13,04%) in USA [20–22], and Italy [26, 27, 36] two (8.69%) in Spain [23, 24], China [12, 25], and India [28, 29], and for the rest of the countries mentioned, one (4.34%) publication was found per country (Turkey [30], United Kingdom [31], Australia [32], Poland [33], Egypt [34], and Korea [35]). (Table 2).

**Table 2:** Demographical and clinical characteristics of the studies selected for the systematic review

No.	Authors/ Year/ Country	Type of Study/Cases No.	A/G	Extraoral Manifestations	Oral Manifestations
1	Aren G, (2002), Turkey [30]	Case Report 1	7/M	Bilateral facial paralysis, lack of facial expression, blinking, visual problems, speech difficulties, and malformations of the upper and lower limbs	Tongue deformities and dental caries
2	De Serpa Pinto et al., (2002), Brazil [15]	Case Series 12	9 = M, 3 = F, Mean age = 6	12 = Bilateral and unilateral facial paralysis and malformations of the upper and lower limbs	12 = Micrognathia, microstomia, cleft palate, gothic palate, tongue deformities anterior open bite
3	Chungyoon and Zakaria (2003), USA [20]	Case Report 1	18/M	Lack of facial expression, blinking, and visual problems	Periodontal disease, dental caries, and odontogenic abscesses
4	Sensat et al., (2003), USA [21]	Case Report 1	40/M	Bilateral facial paralysis, lack of facial expression, dysphagia, blinking, and visual problems	Microdontia, microstomia, periodontal disease, and dental caries
5	Magalhães et al., (2006), Brazil [16]	Case Series 29	18 = M, 11 = F, Mean age = 1,5	29 = Blinking, visual problems, and malformations of the upper and lower limbs	29 = Micrognathia, tongue deformities, cleft palate, and gothic palate
6	Lima et al., (2008), Brazil [17]	Case Report 1	5/M	Unilateral facial paralysis, lack of facial expression, blinking, and visual problems, speech difficulties, and compromised breathing	Tongue deformities and anterior open bite
7	Scarpelli et al., (2008), Brazil [18]	Case Report 1	5/M	Bilateral facial paralysis and speech difficulties	Micrognathia, gothic palate, tongue deformities, dental caries, Class II malocclusion, and anterior open bite
8	Escoda-Francolí et al., (2009), Spain [23]	Case Report 1	49/F	Malformations of the upper and lower limbs	Microstomia, total edentulism, and cleft palate
9	Cai et al., (2012), China [25]	Case Series 3	2 = M, 1 = F, Mean age = 19,3	2 = Bilateral facial paralysis, blinking, and visual problems 1 = Unilateral facial paralysis, blinking, and visual problems	3 = Labial incompetence, anterior open bite with mandibular hyperplasia, crowded dentition, tongue deformities
10	Guijarro-Martínez et al., (2012), Spain [24]	Case Report 1	15/F	Lack of facial expression and speech difficulties	Labial incompetence, anterior open bite tongue deformities, reduced vestibular depth in the upper and low lip, and periodontal disease
11	Bianchi et al., (2013), Italy [26]	Case Report 1	23/M	Bilateral facial paralysis	Micrognathia, and Class II malocclusion
12	Greene et al., (2015), United Kingdom [31]	Care Report 1	12/M	Speech difficulties	Velopharyngeal dysfunction
13	Pradhan et al., (2015), Australia [32]	Case Report 1	19/F	Lack of facial expression	Dental caries
14	Magnifico et al., (2017) Italy [27]	Case Report 1	23/M	Bilateral facial paralysis, blinking, and visual problems	Micrognathia, labial incompetence, and crowded dentition
15	Magnifico et al., (2017) Italy [36]	Case series 58	NI / Mean age = 10	50 = Strabismus, absence of blinking 24 = Malformations of the upper and lower limbs 28 = Reduced TMJ movements	42 = Tongue deformities 8 = Lip and palatal cleft 40 = Labial incompetence
16	Mahrous et al., (2018), USA [22]	Case Report 1	40/F	Bilateral facial paralysis, blinking, visual problems, and malformations of the upper and lower limbs	Micrognathia, complete edentulism, reduced vestibular depth, microstomia, tongue deformities,
17	Cudzilo and Brzozowska, (2019), Poland [33]	Case Report 1	9/F	Bilateral facial paralysis and lack of facial expression	Crowded dentition, hypoplastic enamel, and periodontal disease
18	Freire et al., (2019), Brazil [19]	Case Report 1	5 months/F	Lack of facial expression, blinking, and visual problems	Micrognathia, microstomia, and tongue deformities
19	Hassib et al., (2020), Egypt [34]	Case Series 4	4 = M Mean age = 3,8	4 = Lack of facial expression, blinking, visual problems, and malformations of the upper and lower limbs	4 = Gothic palate, dental caries, periodontal disease, tongue deformities, microdontia, enamel hypoplasia, premature eruption
20	Chen et al., (2021), China [12]	Case report 1	21/M	Lack of facial expression, blinking, and visual problems	Microstomia, crowded dentition, periodontal disease, dental caries, and gothic palate
21	Lee and Moon, (2022). Korea [35]	Case Report 1	7/M	Blinking, visual problems, and malformations of the upper and lower limbs	Microstomia, tongue deformities, anterior deep bite
22	Duggal et al., (2023), India [28]	Case Report 1	9/F	Bilateral facial paralysis, lack of facial expression, blinking, and visual problems	Anterior open bite, crowded dentition
23	Mittal et al., (2023), India [29]	Case Report 1	5/M	Hypotonia, malformations of the upper and lower limbs, lack of facial expression, blinking and visual problems, and compromised breathing	Gothic palate, dental caries, periodontal disease, hypoplastic enamel, microstomia, and micrognathia

*Abbreviations: Not information = NI; Number = No.; Age = A; Gender = G; Female = F; Male = M; Moebius syndrome = MS

### Oral and extraoral manifestations of patients with MS

The clinical characteristics of the 124 cases of MS patients are summarized in Table 2. The age of the patients ranged from 0,5 to 49 years, with a mean (DE) of 14,92 *±* 12,80. Males were more affected (64%) with this syndrome, with a male-to-female ratio of 2:1; only one article did not report gender [36]. The most frequent extraoral manifestations were blinking and visual problems (78,22%), malformations of the upper and lower limbs (58,22%), bilateral facial paralysis (12,90%), lack of facial expression (12.09%), and unilateral facial paralysis (6,45%). On the other hand, the most frequent oral manifestations were tongue deformities (78,22%), micrognathia (37,90%), labial incompetence (36,29%), cleft palate (22,87%), gothic palate (16,12%), microstomia (15,32%), anterior open bite (15,32%), dental caries (8,87%), and periodontal disease (8,06%).

### Comprehensive dental management of patients with MS

The integral dental management of the 124 cases of MS patients is summarized in Table 3, and the descriptive statistics are summarized in Table 4. The majority of MS patients were treated by pediatric dentistry (60,86%), using a surgical approach (56,52%), and orthodontic and orthopedic maxillary (43,47%) followed by restorative (39,13%), and periodontal treatments (21,73%).

**Table 3:** Dental management of patients with MS.

No.	Authors	Treatment	Dental Discipline	Follow-up (years)
1	Aren G [30]	Dental extractions Dental restorations	Pediatric dentistry Restorative dentistry Oral and maxillofacial surgery	NI
2	De Serpa Pinto [15]	Periodontal treatment Dental restorations	Pediatric dentistry Restorative dentistry	NI
3	Chungyoon and Zakaria [20]	Periodontal treatment Dental restorations Dental restorations	Pediatric dentistry Periodontics Restorative dentistry Oral and maxillofacial surgery	NI
4	Sensat et al., [21]	Periodontal treatment Dental restorations Dental prostheses	Periodontics Restorative dentistry Prosthodontics	NI
5	Magalhães et al., [16]	Orthopedic appliances	Pediatric dentistry Orthodontic and maxillary orthopedics	8
6	Lima et al., [17]	Frenectomy Removable orthodontic appliance	Oral and maxillofacial surgery Orthodontic and maxillary orthopedic Pediatric dentistry	1
7	Scarpelli et al., [18]	Behavioral therapy Dental restorations	Restorative dentistry Pediatric dentistry	NI
8	Escoda-Francolí et al., [23]	Dental implants	Oral implantology	8
9	Cai et al., [25]	Orthognathic surgery	Oral and maxillofacial surgery	4
10	Guijarro-Martínez et al. [24],	Pre-surgical orthodontics Orthognathic surgery	Orthodontic and maxillary orthopedic Oral and maxillofacial surgery Pediatric dentistry	0.5
11	Bianchi et al., [26]	Dental extractions Orthodontic appliance Orthognathic surgery Smile surgery	Orthodontic and maxillary orthopedic Oral and maxillofacial surgery	5
12	Greene et al., [31]	Orthopedic appliances	Orthodontic and maxillary orthopedic Oral and maxillofacial surgery Pediatric dentistry	1,3
13	Pradhan et al., [32]	Dental extractions Dental restorations	Restorative dentistry Oral and maxillofacial surgery	7
14	Magnifico et al., [27]	Orthodontic appliance Orthognathic surgery Smile surgery	Orthodontic and maxillary orthopedic	4
15	Magnifico et al., [36]	Removable/ fixed orthodontic appliance	Orthodontic and maxillary orthopedic	NI
16	Mahrous et al., [22]	Implant-supported dentures	Oral implantology Prosthodontics	0.6
17	Cudzilo and Brzozowska, [33]	Dental extractions Removable / fixed orthodontic appliance	Orthodontic and maxillary orthopedic Oral and maxillofacial surgery Pediatric dentistry	NI
18	Freire et al., [19]	Frenectomy	Pediatric dentistry Oral and maxillofacial surgery	The patient had died before reaching one year of age of a reported cardiac arrest.
19	Hassib et al., [34]	Dental extractions Periodontal treatment Dental restorations Root canal treatment Dentures	Pediatric dentistry Periodontics Restorative dentistry Oral and maxillofacial surgery Prosthodontics	NI
20	Chen et al., [12]	Dental extractions Periodontal treatment Dental restorations Root canal treatment Periapical surgery	Periodontics Restorative dentistry Oral and maxillofacial surgery	2
21	Lee and Moon, [35]	Removable / fixed orthodontic appliance	Orthodontic and maxillary orthopedic Pediatric dentistry	9,4
22	Duggal et al. [28],	Dental extractions Orthodontic camouflage	Pediatric dentistry Orthodontic and maxillary orthopedic	1
23	Mittal et al., [29]	Dental extractions Periodontal treatment Dental restorations Root canal treatment Dentures	Pediatric dentistry Periodontics Restorative dentistry Oral and maxillofacial surgery Prosthodontics	0.6

*Abbreviations: Not information = NI; Moebius syndrome = MS

**Table 4:** Summary of clinical data of MS patients

Variables	Values	%
**Articles**	23	
**Total Cases**	124	
**Age (years)**		
< 15 ≥ 15	112 12	90.32 10.68
Mean ± SD Range (Min-Max)	14.92 ± 12.80 0.5–49	
**Gender**		
Male Female	42 24	64.00 36.00
**Extraoral Manifestations**		
Hypotonia	1	0.80
Speech difficulty Bilateral facial paralysis Unilateral facial paralysis Compromised breathing Lack of facial expression Blinking and visual problems Malformations of the upper and lower limbs	5 16 8 2 15 97 73	4.03 12.90 6.45 1.61 12.09 78.22 58.87
**Oral Manifestations**		
Cleft palate Dental caries Microstomia Microdontia Micrognathia Dental crowding Anterior open bite Gothic palate Periodontal disease Tongue deformities Premature eruption Hypoplastic enamel Labial incompetence Class II Malocclusion Total/partial edentulism Reduced vestibular depth Mandibular/Maxilla hyperplasia	42 11 19 2 47 7 19 20 10 97 4 2 45 2 2 2 1	33.87 8.87 15.32 1.61 37.90 5.64 15.32 16.12 8.06 78.22 3.22 1.61 36.29 1.61 1.61 1.61 0.80
**Dental Discipline**	***n*** **(23)**	
Periodontics Prosthodontics Pediatric dentistry Oral implantology Restorative dentistry Oral and maxillofacial surgery Orthodontic and maxillary orthopedic	5 4 14 2 9 13 10	21.73 17.39 60.86 8.69 39.13 56.52 43.47
**Follow-up (years)**		
Mean ± SD	3.74 ± 3.22	

Data were reported as mean ± standard deviation and *n* (%). *Abbreviations: Moebius syndrome=MS

### Evaluation of the methodological quality of the selected studies

Tables 5 and 6 show the results of the quality assessment of the included studies. Based on the checklist used to rate the articles, all studies achieved total scores [12, 15–36], resulting in a low risk of bias in all selected studies.

**Table 5:** Results of the quality assessment for case reports

No.	Authors	Q1	Q2	Q3	Q4	Q5	Q6	Q7	Q8	Overall Score and Quality
1	Aren G [30]	**Y**	**Y**	**Y**	**Y**	**Y**	**N**	**Y**	**Y**	**87.5**
2	Chungyoon and Zakaria [20]	**Y**	**Y**	**Y**	**Y**	**Y**	**N**	**Y**	**Y**	**87.5**
3	Sensat *et al.*, [21]	**Y**	**Y**	**Y**	**Y**	**Y**	**Y**	**Y**	**Y**	**100**
4	Lima *et al.*, [17]	**Y**	**Y**	**Y**	**Y**	**Y**	**Y**	**Y**	**Y**	**100**
5	Scarpelli *et al.*, [18]	**Y**	**Y**	**Y**	**Y**	**U**	**N**	**Y**	**Y**	**75**
6	Escoda-Francolí *et al.*, [23]	**Y**	**Y**	**Y**	**Y**	**Y**	**Y**	**Y**	**Y**	**100**
7	Guijarro-Martínez *et al.*, [24]	**Y**	**Y**	**Y**	**Y**	**Y**	**Y**	**Y**	**Y**	**100**
8	Bianchi *et al.*, [26]	**Y**	**Y**	**Y**	**Y**	**Y**	**Y**	**Y**	**Y**	**100**
9	Greene *et al.*, [31]	**Y**	**Y**	**Y**	**Y**	**Y**	**Y**	**Y**	**Y**	**100**
10	Pradhan *et al.*, [32]	**Y**	**Y**	**Y**	**Y**	**Y**	**Y**	**Y**	**Y**	**100**
11	Magnifico *et al.*, [27]	**Y**	**Y**	**Y**	**Y**	**Y**	**Y**	**Y**	**Y**	**100**
12	Mahrous *et al.*, [22]	**Y**	**Y**	**Y**	**Y**	**Y**	**Y**	**Y**	**Y**	**100**
13	Cudzilo and Brzozowska, [33]	**Y**	**Y**	**Y**	**Y**	**Y**	**N**	**Y**	**Y**	**87.5**
14	Freire *et al.*, [19]	**Y**	**Y**	**Y**	**Y**	**Y**	**Y**	**Y**	**Y**	**100**
15	Chen *et al.*, [12]	**Y**	**Y**	**Y**	**Y**	**Y**	**Y**	**Y**	**Y**	**100**
16	Lee and Moon, [35]	**Y**	**Y**	**Y**	**Y**	**Y**	**Y**	**Y**	**Y**	**100**
17	Duggal *et al.*, [28]	**Y**	**Y**	**Y**	**Y**	**Y**	**Y**	**Y**	**Y**	**100**
18	Mittal *et al.*, [29]	**Y**	**Y**	**Y**	**Y**	**Y**	**Y**	**Y**	**Y**	**100**

**Question (Q); N/A, not aplicable; Y, yes; N, no; U, unclear**

Q1: Were patient’s demographic characteristics clearly described?

Q2: Was the patient’s history clearly described and presented as a timeline?

Q3: Was the current clinical condition of the patient on presentation clearly described?

Q4: Were diagnostic tests or assessment methods and the results clearly described?

Q5: Was the intervention (s) or treatment procedure (s) clearly described?

Q6: Was the post-intervention clinical condition clearly described?

Q7: Were adverse events (harms) or unanticiped events identified and described?

Q8: Does the case report provide takeaway lessons?

**Table 6:** Results of the quality assessment for cases series

No.	Authors	Q1	Q2	Q3	Q4	Q5	Q6	Q7	Q8	Q9	Q10	Overall Score and Quality
19	De Serpa Pinto *et al.*,[15]	**Y**	**Y**	**Y**	**Y**	**Y**	**Y**	**Y**	**Y**	**Y**	**Y**	**100**
20	Magalhães *et al.*, [16]	**Y**	**Y**	**Y**	**Y**	**Y**	**Y**	**Y**	**Y**	**Y**	**Y**	**100**
21	Cai *et al.*, [25]	**Y**	**Y**	**Y**	**Y**	**Y**	**Y**	**Y**	**Y**	**Y**	**Y**	**100**
22	Hassib *et al.*, [34]	**Y**	**Y**	**Y**	**Y**	**Y**	**Y**	**Y**	**Y**	**Y**	**Y**	**100**
23	Magnifico *et al.*, [36]	**Y**	**Y**	**Y**	**Y**	**Y**	**Y**	**Y**	**Y**	**Y**	**Y**	**100**

**Question (Q); N/A, not applicable; Y, yes; N, no; U, unclear**

Q1: Were there clear criteria for inclusion in the case series?

Q2: Was the condition measured in a standard, reliable way for all participants included in the case series?

Q3: Were valid methods used for the identification of the condition for all participants included in the case series?

Q4: Did the case series have consecutive inclusion of participants?

Q5: Did the case series have consecutive inclusion of participants?

Q6: Was there clear reporting of the demographics of the participants in the study?

Q7: Was there reporting of clinical information of the participants?

Q8: Were the outcomes or follow-up results of cases clearly reported?

Q9: Was there clear reporting of the presenting sites(s)/clinics(s) demographic information?

Q10: Was statistical analysis appropriate?

## Discussion

The present systematic review analyzed clinical studies, mainly case reports and case series, emphasizing the comprehensive dental treatment of patients with MS.

MS is a rare, congenital, non-progressive, neuropathological condition that affects the development and function of the abducens and facial nerves, involving other cranial nerves such as III, IV, V, IX, X, and XII [37]. A recent systematic review, which comprises the most extensive series of cases with MS (*n* = 449), presented evidence of the existence of two groups of patients: Group 1, with a strong association between micrognathia, limb anomalies, and swallowing difficulties, and Group 2; phenotypically more diverse but associated with radiologically detectable neurological anomalies and developmental delay [38].

This study found that upper and lower extremity malformations, visual and blinking problems, and bilateral facial paralysis were the most frequent extraoral findings in almost all patients with MS. Thus, our findings agree with what has been reported in the literature. A cross-sectional study evaluating the prevalence of upper extremity malformations in 25 patients with MS showed that the most frequent were syndactyly (32%), brachysyndactyly (20%), and amniotic band syndrome (12%) [39]. Regarding ocular manifestations, another study found a higher prevalence of esotropia, abduction limitation, and compound hypermetropic astigmatism in patients with this syndrome [40]. On the other hand, a prospective clinical study that analyzed 25 patients with MS aged between 2 months and 54 years showed that the most frequent extraoral manifestations were speech problems (68%), feeding difficulties in infancy (64%), bilateral facial paralysis (64%), unilateral facial paralysis (32%) and drooling (32%). Whereas the most frequently observed orofacial anomalies were tongue dysfunction and anomalies (64%), micrognathia (32%), microglossia (28%), cleft palate (16%), and cleft lip (4%) [41]. Thus, based on these first findings, it is essential to achieve an accurate early diagnosis and apply a multidisciplinary treatment approach with long-term follow-up, which not only helps to overcome the challenges of treatment but can also reduce the impact of sequelae on the lives of patients and their families, providing great psychosocial well-being benefits.

Interestingly, 16 (73%) studies identified bilateral facial paralysis as the most prevalent extraoral manifestation of MS, while 11 (50%) studies reported unilateral facial paralysis, the latter being rarer. Some cases did not report this sign on physical examination, probably due to the specific focus on oral manifestations and dental treatment; however, facial paralysis is a characteristic sign of the disease [4–6].

Patients with MS present with congenital facial paralysis characterized by facial nerve damage and may also be associated with abducens nerve paralysis, resulting in impaired eye movement. This paralysis can be unilateral, bilateral, complete, partial, symmetrical, or asymmetrical [8]. Congenital facial paralysis has far-reaching psychological and functional consequences [10]. On the one hand, the inability to replicate facial expressions together with speech difficulties leads to the fact that individuals with MS may be perceived as unfriendly and unintelligent. This leads to limited social interactions, negatively affecting the individual’s psychological and social development [42]. In this sense, it has been shown that individuals with MS have a less sensitive parasympathetic system during the observation of social stimuli compared to individuals without the syndrome; this highlights the importance of studying autonomic responses in different social contexts, where decreased autonomic activity in response to the observation of others’ facial expressions could, at least in part, explain some of the difficulties experienced by individuals with MS during social interventions [43]. On the other hand, a clinical study demonstrated that some areas of psychosocial adjustment, such as behavior, anxiety, depression, low overall life satisfaction, with low success orientation and high incidence of suicidal thoughts, were more accentuated in individuals with MS, compared to the general population [44, 45]. This highlights the importance of implementing programs to enhance oral-motor and speech training and thereby improve patients’ quality of life. These include therapies for breathing control (meditation and relaxation), massage, and neuromuscular training, accompanied by psychological and speech therapy sessions. On the other hand, the functional sequelae of congenital facial paralysis include incomplete ocular closure that can generate corneal exposure and lead to blindness and convergent strabismus; difficulties in eating and drinking, including cheek bagging, as well as severe drooling; hearing and speech problems; lack of muscle contraction that can alter palatogenesis and produce cleft palate or palate gothic; and micrognathia [27].

Concerning the oral findings in MS, a great variety of atypical features have been described. In the present review, we found a higher frequency of oral alterations such as micrognathia, anterior open bite, microstomia, cleft palate, gothic palate, malocclusions (skeletal class II), tongue deformities, dental crowding, dental caries, periodontal disease (gingivitis and/or periodontitis) and even self-inflicted oral trauma [46].

For didactic purposes and to further elaborate on the comprehensive dental management of MS, the discussion was divided into subtopics emphasizing those treatments performed together or go hand in hand, as shown below.

### Surgical, orthodontic, and orthopedic treatment of MS

Eighteen (78,26%) studies reported on the different surgical, orthodontic, and orthopedic treatment protocols to resolve the prominent dental-skeletal anomalies and thereby allow long-term stability in the MS patient [16, 17, 24–28, 31, 33, 35]. The most common craniofacial malformation present in patients with MS, which is usually evident from birth, is micrognathia [16, 18, 19, 22, 26, 27, 29]. In individuals with normal craniofacial development, the maxilla grows anteroinferiorly. Still, in subjects with MS, the maxilla exhibits excessive growth in the anterior direction due to decreased muscle activity of the upper lip (orbicularis oris muscle of the mouth) and, in addition, its growth in the inferior direction is arrested [12, 15, 21]. This makes the lip seal deficient, producing lip incompetence, which is closely related to skeletal class II malocclusion and anterior open bite [19]. Therefore, with occlusal insufficiency, other severe and significant disorders occur, such as deficiencies in chewing and speech and facial esthetics being severely compromised; hence, aesthetic improvement is required [13]. Combined treatment is essential in these cases, where specialists in the surgical area, orthodontics, and maxillary orthopedics can participate [16–19]. The treatment will depend on the severity of the case, but it is always important to approach it from an interdisciplinary point of view for the patient’s good [20–22]. In highly severe dentofacial deformities, patients require multiple treatments such as pre- and post-surgical orthodontics, orthopedic appliances, orthognathic surgery followed by soft tissue management, and smile surgery [27].

In the present study, 97 (78,22%) MS patients required orthodontic and orthopedic treatment to correct their dentofacial deformities (micrognathia, soft palate weakness associated with glossoptosis, gothic palate, and lip incompetence problems). In most studied patients, a tremendous functional and esthetic improvement was demonstrated, positively affecting their quality of life and their families. Some patients with MS (41,93%) could not benefit from such therapy. It is essential to mention that orthodontic and orthopedic treatment can be complicated for both the clinician and the patient. This has to do with some technical problems, such as the process of taking impressions with alginate or some other impression biomaterial to obtain the study/working models and the subsequent fabrication and adaptation of the fixed/removable appliances, which is usually tedious and sometimes difficult due to the same condition of the patients [47]. In addition, other reasons patients could not adhere to orthodontic therapy are socioeconomic status, i.e., patients with low income, those who live in cities far from dental rehabilitation centers, and those who have difficulty with transportation. Also, some patients have nasogastric tubes, so the devices are inaccessible. In addition, the knowledge that patients have to continue treatment into adolescence and, in cases of orthognathic surgery, after surgical procedures is a discouraging factor for parents/guardians [6].

Before placing fixed/removable orthodontic appliances, it is essential to consider that patients with MS have microstomia and other characteristics such as restricted tongue muscle movements, dry mucous membranes, and angular cheilitis, which complicates dental therapy. In addition, the use of these devices can cause periodontal disease and dental caries, which is attributed both to the presence of these devices and to the fact that patients with MS have malformations in their hands and fingers, which can cause problems with oral hygiene. Therefore, preventive therapy is vital in these patients through the periodic application of fluoride, oral hygiene instructions to the patient’s parents or guardian, and ultrasonic prophylaxis at least every 4 to 6 months [24–28, 31].

Orthognathic surgery by mandibular advancement is the treatment of choice for micrognathia in young adults with MS [25, 27]. On the other hand, in pediatric patients, micrognathia can lead to airway obstruction, especially during sleep, which compromises the infant’s life. These patients usually require tracheostomy; however, another therapeutic alternative could be distraction osteogenesis (DO), mainly in children, young patients, and/or young adults, due to the high capacity of osteogenesis and also because pre-surgical orthodontic treatment is usually started at an early age and completed in adulthood [48]. Contrary to this, some successful cases of patients > 30 years of age have also been reported using these devices and achieving bone augmentation up to 25 mm in length for five years [49, 50]. However, DO shows some surgical drawbacks or limitations, such as the presence of wound site infections, prolonged hospital stays, and intense relapses associated with considerable advances (> 10 mm) due to high perimandibular soft tissue tension [51].

Smile surgery by free muscle transfer using the gracilis muscle and nerves is a safe and reliable technique for facial reanimation with excellent aesthetic and functional results. This surgical procedure is performed by plastic surgery in conjunction with oral and maxillofacial surgery. Usually, after this surgical procedure, facial mimicry is restored, so patients with SM can implement a new smile motor circuit [25]. For this reason, this procedure should be performed as soon as possible to reduce the psychological consequences of the syndrome and, on the other hand, to improve the patient’s interpersonal relationships and psychophysical development [26].

### Restorative, periodontal, and prosthetic treatment of MS

Dental caries and periodontal disease (gingivitis and periodontitis) are two other highly prevalent conditions that affect oral health in patients with MS. The formation of dental caries in patients with MS has been attributed mainly to the use of a high-carbohydrate (cariogenic) diet [52]. In addition, it has been shown that these patients have a reduced and altered salivary composition, i.e., patients with MS have a decreased salivary flow, buffering capacity, and α-amylase activity, creating a more susceptible environment that favors bacterial colonization [36]. This, combined with reduced activity of the muscles of the perioral region, results in the appearance of early carious lesions. In most cases, treatment consisted of carefully removing the carious lesions and restoring the teeth using different biomaterials such as amalgams and resins. Some other authors reported the placement of chromium steel crowns. Unquestionably, the choice of biomaterials will depend on the dentist’s decision based on the clinical scenario. In this regard, placing more durable materials in the mouth is recommended to avoid recurrent appointments for changing restorations. Composite resin restorations have less longevity and more secondary caries than amalgam restorations [53, 54].

As mentioned above, periodontal disease in MS patients is mainly caused by the use of orthodontic/orthopedic appliances and poor oral hygiene. It is still unknown whether patients with MS are more genetically predisposed than the general population to periodontal disease. The present study treated patients by scaling and root planing, prophylaxis, education, and oral hygiene instructions, significantly improving periodontal health. Future studies would be advisable to determine the composition of the periodontal microbiota in these patients, to know the cytokine profile involved in the immune response and to compare it with healthy patients, as well as to study some genetic variants that could be risk factors or genetic protectors against the development of periodontal disease [55, 56]. .

Severe periodontal problems can result in tooth loss [57]. Partial or total edentulism is also characteristic of SM [22, 23]. Prosthetic treatment represents a significant challenge for the dentist, so it is crucial to consider the following: poor neuromuscular control (facial paralysis), accompanied by small mouth opening and speech difficulties, can make prosthetic therapy difficult [11]. In the present study, three patients were rehabilitated with removable partial dentures [22, 29, 34] and two others with implant-supported complete dentures [22, 23]. The only inconvenience reported was concerning the primary impression taking, so a special tray was fabricated to fit perfectly to the size of the patient’s mouth; the rest of the prosthodontic procedures were performed conventionally [34].

In reality, the treatment of these conditions is very similar to that of healthy patients. The difference lies in the fact that MS patients are not very cooperative due to the anxiety of the situation and sometimes have violent behavior, which could further complicate a simple dental procedure. For this reason, some patients receive general anesthesia or are treated under sedation while undergoing full oral rehabilitation. The main problem that arises when administering general anesthesia to these patients is complex airway management. It is important to remember that patients with MS presenting with severe micrognathia and microstomia can make ventilation and mask intubation difficult. In addition, this syndrome frequently leads to respiratory failure and dysphagia due to cranial nerve palsies, resulting in an increased risk of postoperative complications [58, 59].

This review has some limitations, such as the small number of articles that evaluate oral manifestations and comprehensively approach the dental management of patients with MS. In addition, some articles did not describe the follow-up of patients after dental treatment.

## Conclusions

MS is a rare neuropathological disorder that affects the development and function of the abducens and facial nerves. Its etiology is idiopathic; however, some genetic and in-utero vascular factors have been attributed to it. MS presents a combination of craniofacial, ophthalmologic, dental, and orthopedic conditions of particular interest; therefore, its treatment implies a multidisciplinary approach.

From the results presented in this systematic review, we can conclude the following:

- The most frequent extraoral findings of MS are blinking and visual problems, malformations of the upper and lower limbs, bilateral facial paralysis, lack of facial expression, and unilateral facial paralysis.
- The most representative intraoral findings of MS are tongue deformities, micrognathia, labial incompetence, cleft palate, gothic palate, microstomia, anterior open bite, dental caries, and periodontal disease, which have become a great challenge for management by the dentist since it involves the participation of different specialists in the area.
- Thus, patients with MS are subjected to different surgical procedures, orthodontic and orthopedic treatments, and restorative procedures to resolve their problems.
- A comprehensive dental treatment plan adapted to each patient is required, covering both the resolution of the problem and the maintenance of oral health.

## Data Availability

No datasets were generated or analysed during the current study.

## Abbreviations

MS: Moebius syndrome
DO: Distraction osteogenesis
